# Organizing Pneumonia After Exposure to Sodium Hypochlorite: A Case Report

**DOI:** 10.7759/cureus.12025

**Published:** 2020-12-11

**Authors:** Inês Santos, Sandra Lucas, Rui Seixas, Ireneia Lino

**Affiliations:** 1 Internal Medicine, Hospital do Espírito Santo de Évora E.P.E, Largo Senhor da Pobreza, 7000-811, Évora, PRT

**Keywords:** corticosteroid-sparing agent, organizing pneumonia, interstitial lung disease, sodium hypoclorite, transbronchial lung biopsy, corticosteroid therapy

## Abstract

Organizing pneumonia (OP) is a rare inflammatory lung disease with a difficult diagnosis and sparse mentions in the literature. In most cases, the etiology is unknown but may be associated with infections, systemic disorders, exposure to industrial toxins and environmental pollutants, or even drug toxicity.

This report describes a 77-year-old male who presented to the hospital with nonproductive cough, myalgias, fever, and progressive weight loss after prolonged exposure to sodium hypochlorite. The patient was treated with multiple courses of antibiotics with no pathogen isolation. Chest CT revealed condensation foci of all pulmonary lobes. His clinical history, laboratory results, and CT images led to the diagnosis of OP, which was confirmed with a transbronchial lung biopsy. The patient was treated with oral prednisolone with clinical improvement and discharge one week after the beginning of the corticosteroid treatment.

OP diagnosis can be challenging and, if not considered, may lead to a delay in providing appropriate treatment to the patients, which can often lead to a prolonged hospital stay and poor outcomes.

## Introduction

Organizing pneumonia (OP), formerly known as bronchiolitis obliterans organizing pneumonia (BOOP), is a disorder with distinctive clinical, radiological, and pathological features. It usually appears as a clinical syndrome in individuals in their fifth or sixth decades of life without any predilection for gender or race. One study has shown a higher prevalence among non-smoking individuals [1].

This lung condition is a type of diffuse interstitial lung disease characterized by fibroblastic plugs in the lumen of the respiratory bronchioles, alveolar ducts, and alveoli [2]. It can be classified into two types: cryptogenic and secondary OP. This latter one is associated with various underlying conditions, including previous infection, organ transplantation, radiotherapy, systemic disorders like connective tissue diseases, inflammatory bowel disease, and fibrosing lung disease, drug toxicity, exposure to industrial toxins, and environmental pollutants [3]. Most cases of OP reported so far have been cryptogenic [4]. The majority of patients present with subacute symptoms such as fever, malaise, and a cough that is generally persistent and nonproductive. Patients may also present with dyspnea and weight loss [5]. Less common symptoms include pleuritic pain and hemoptysis [6].

There is little information published on the effects of inhalation of sodium hypochlorite; however, its severe effects may include hypoxia, pneumonitis, tracheobronchitis, pulmonary edema, and respiratory failure [7].

## Case presentation

A 77-year-old white male with type 2 diabetes mellitus and hypertension presented to the emergency department with one week of nonproductive cough, myalgias, fever, and weight loss. He had taken amoxicillin/clavulanic acid, which had been prescribed for seven days, with no clinical benefit. The patient reported that symptoms had started after intensive cleaning of his holiday house, where he had used bleach (sodium hypochlorite), for several days.

**Figure 1:**
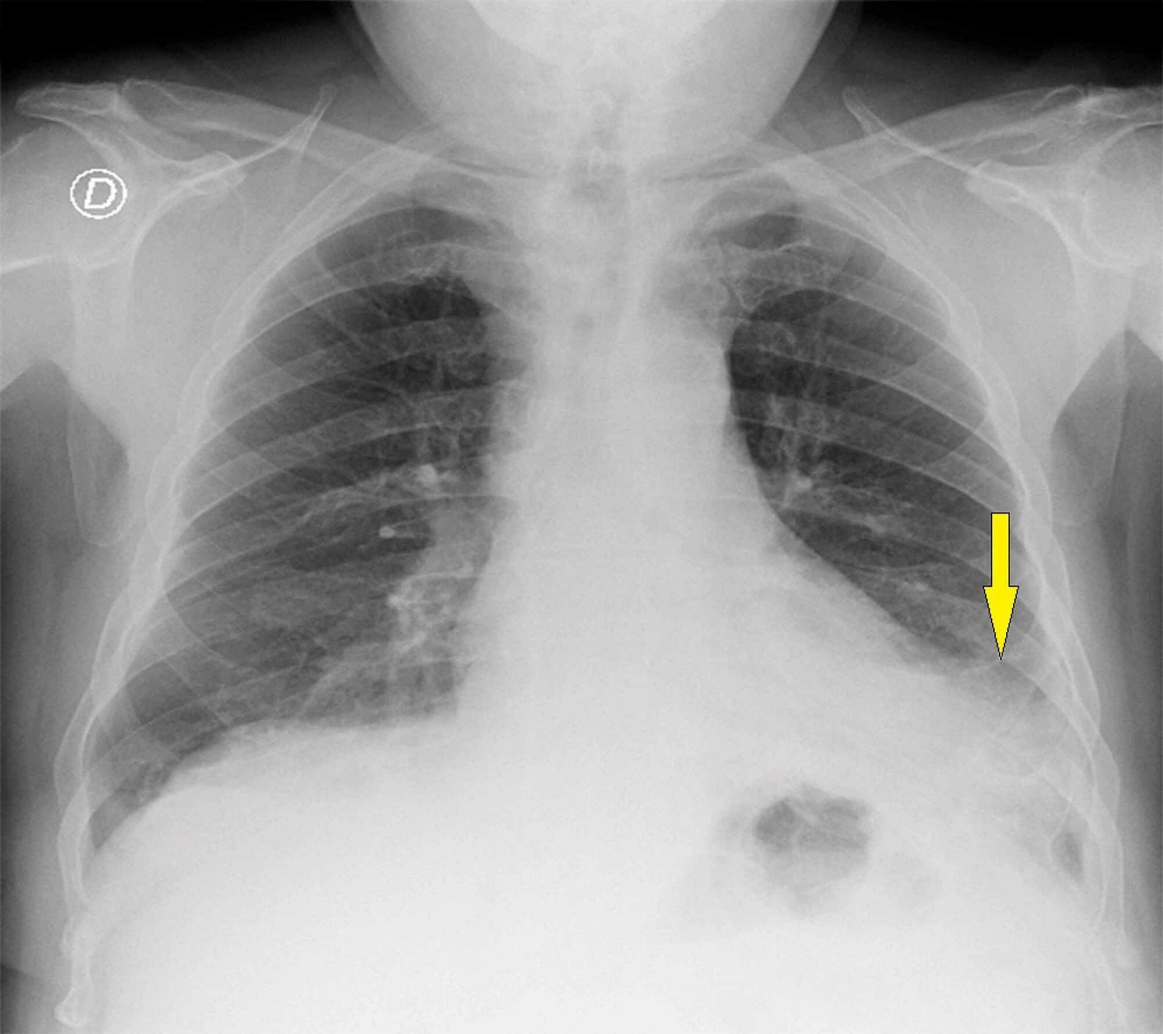
Chest radiography on admission The image shows pulmonary infiltrate in the left lower lung lobe (arrow)

On admission, the physical examination identified fever (38.2 ºC) and bilateral crackles in both lower lung fields in the pulmonary auscultation, without any other remarkable signs. The first laboratory investigation showed a white cell count of 12.0xµL, neutrophil count of 8.7xµL, C- reactive protein of 16.7 mg/dL, creatinine of 1.65 mg/dL, N-terminal pro-B-type natriuretic peptide (NT-pro-BNP) of 160 pg/ml, and ferritin of 1,410 ng/mL. Arterial blood gas analysis on room air showed an arterial oxygen partial pressure (PaO_2_) of 65 mmHg, arterial carbon dioxide partial pressure (PaCO_2_) of 35 mmHg, oxygen saturation of 94%, and lactate of 3.9 mmol/L. Chest radiography revealed an infiltrate in the left lung lobe (Figure 1).

The patient was admitted to the internal medicine department with a diagnosis of community-acquired pneumonia and was started on antimicrobial therapy with levofloxacin.

**Figure 2:**
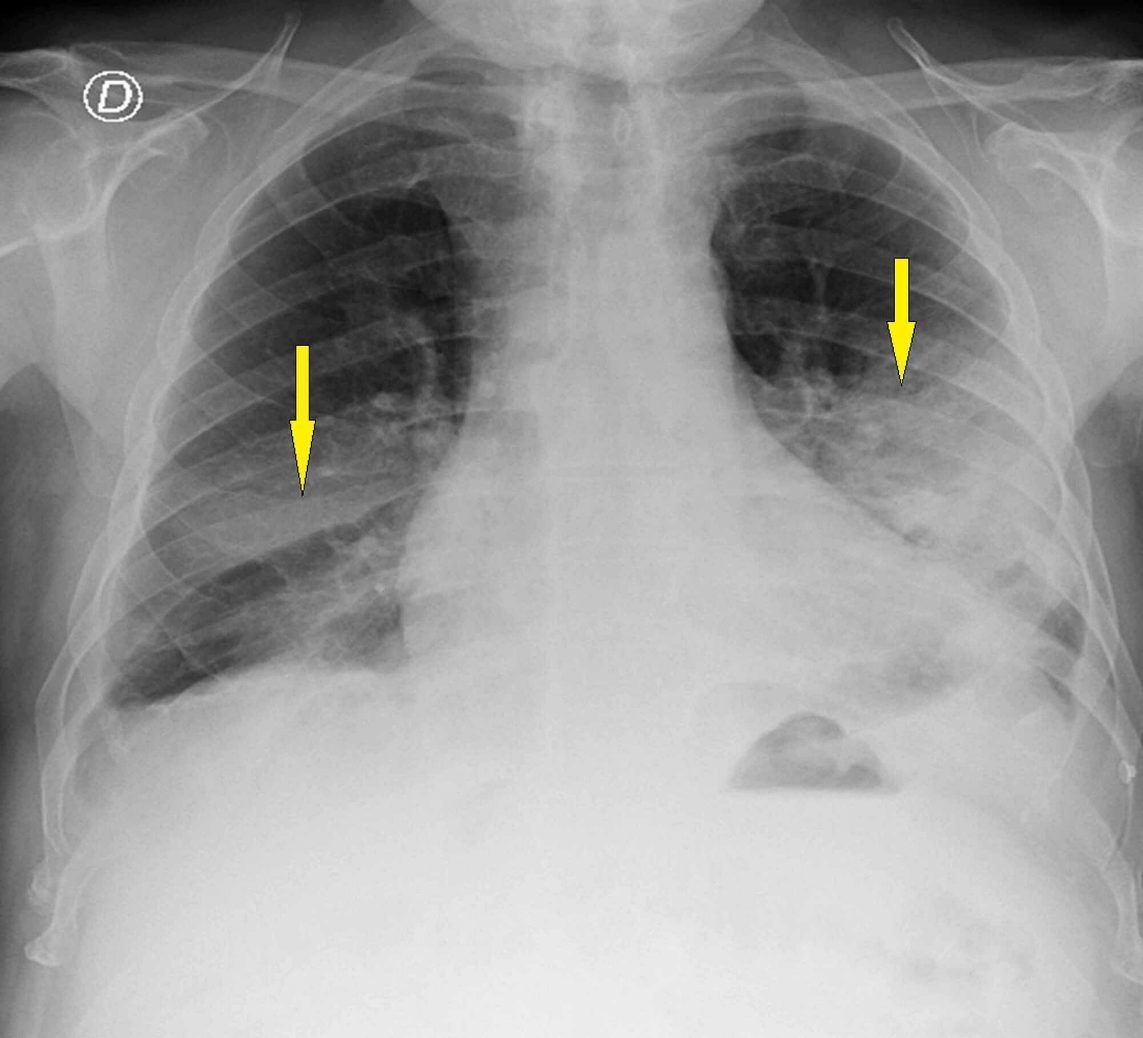
Repeat chest radiography on day five of the hospital stay The image shows bilateral infiltrates involving predominantly the middle and lower lung fields (arrows)

During the hospital stay, the patient had progressive clinical worsening with ongoing fever and an increase in oxygen needs. Chest radiography was repeated on day five of the hospital stay, which revealed worsened imaging with bilateral infiltrates involving predominantly the middle and lower lung fields (Figure 2).

Considering the hypothesis of nosocomial infection and clinical instability, the patient received treatment with broad-spectrum antibiotics with meropenem and vancomycin, but without any clinical improvement.

The urine *Streptococcus pneumoniae* and *Legionella pneumophila* antigen tests were performed; serologies for *Mycoplasma pneumoniae, Borrelia burgdorferi*,* Brucella* spp. and *Chlamydophila pneumoniae* serologies, and microbial analysis of sputum and blood cultures were also done. All the results were negative. Viral pneumoniae was also considered, requesting a respiratory virus panel and severe acute respiratory syndrome coronavirus 2 (SARS-CoV-2) test, which were also negative. The procalcitonin value was 0.14 ng/ml. Bronchofibroscopy was unremarkable and bronchoalveolar lavage culture did not identify any pathogen.

An autoimmune disorder with pulmonary involvement was also considered. Antinuclear antibodies (ANA) and antineutrophil cytoplasmic antibodies (ANCA) were negative. The sedimentation rate was 69 mm/h, and the angiotensin-converting enzyme level was 28 U/L.

**Figure 3:**
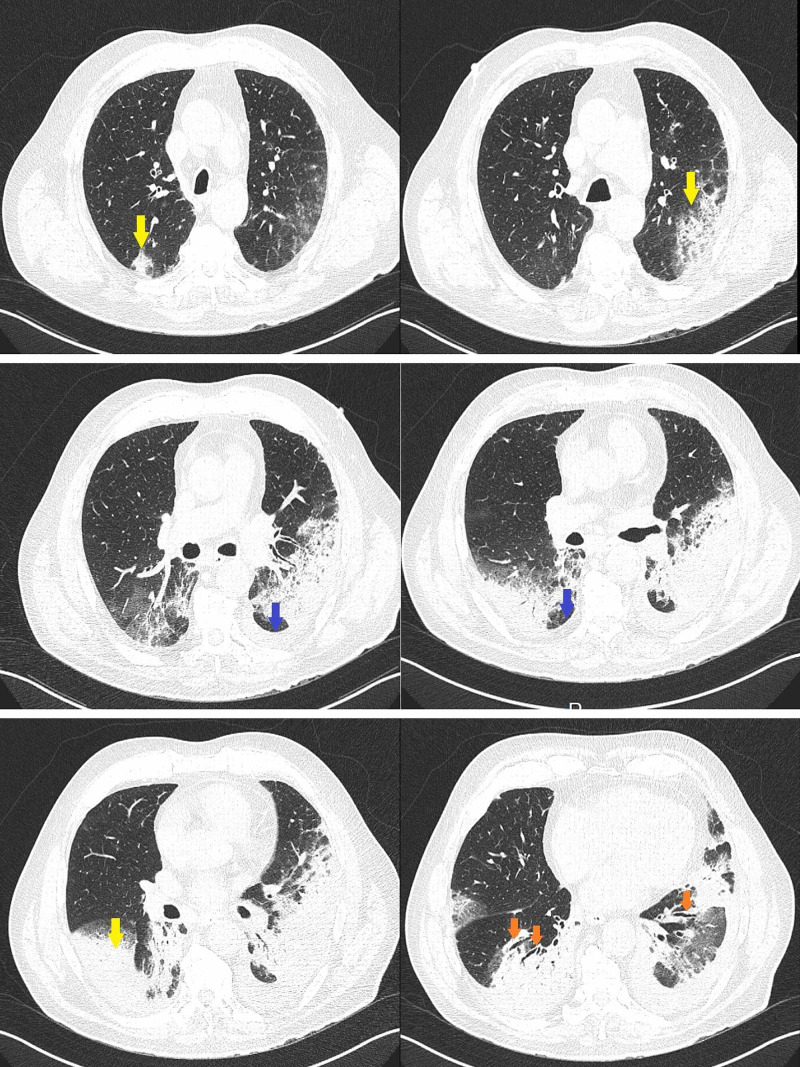
Chest CT images The images show bilateral consolidation, more extensive in lower lobes (yellow arrows) with air bronchogram sign (orange arrow), and bilateral small amount of pleural effusion (blue arrow) CT: computed tomography

In order to better characterize the pulmonary lesions, a high-resolution chest CT was performed and revealed condensation foci of all pulmonary lobes, more extensive in the lower lobes and the posterior segment of the upper left lobe, with some degree of parenchyma distortion. It also showed hilar and mediastinal adenopathies and a small volume of bilateral pleural effusion (Figure 3).

Pulmonary function tests were not performed since the patient's clinical condition did not allow them. Due to the lack of improvement with multiple antimicrobial courses and the absence of pathogen identification, we suspended the antibiotics and the hypothesis of OP was raised.

The patient underwent a transbronchial lung biopsy, which showed an enlargement of the interalveolar septa, fibroblastic buds, and a dispersed inflammatory infiltrate, predominantly lymphocytes and histiocytes, with occasional fibrin deposits, which confirmed the diagnosis of bronchiolitis obliterans. After excluding infectious diseases and while waiting for the biopsy result, the patient was started on corticosteroid therapy with prednisolone 1 mg/Kg/day with progressive clinical improvement. The patient's supplementary oxygen need progressively improved, and he was discharged home after one week of corticotherapy with recommendations to avoid exposure to sodium hypochlorite.

The patient continued corticosteroid therapy for four weeks, which had to be stopped since he manifested a psychotic flare, associated with high-dose corticosteroids. Antipsychotic therapy was started with olanzapine, and a fast withdrawal of corticosteroid was done. The patient revealed mood stabilization without clinical or radiological relapse.

## Discussion

Our patient had been exposed to sodium hypochlorite for a long period of time and it was the trigger for his medical condition.

There is scarce information available on the effects of inhalation of hypochlorite, but the extent of injury depends on both the duration and the concentration of exposure [7]. Severe respiratory effects such as upper airway swelling, hypoxia, pneumonitis, stridor, pulmonary edema, and respiratory failure have been described [7]. One proposed mechanism is that hypochlorite could release chlorine that causes damage to moist tissues by reacting with water to form hydrochloric and hypochlorous [7]. These products can disrupt cellular proteins and cause cytotoxic damage to the cells of the respiratory tract [7]. Alveolar epithelial injury is followed by leakage of plasma proteins, recruitment of fibroblasts, and fibrin formation within the alveolar lumen [8].

OP is classified as an interstitial lung disease that affects the small airways [2]. OP can be differentiated from the other interstitial lung diseases because it predominates an inflammatory pathway rather than a fibrosis pathway. There is abundant capillarization in the intra-airway fibromyxoid lesions due to vascular growth factors, compared with minimal vascularization in other interstitial lung diseases [4]. On the other hand, apoptotic activity is also higher in OP, suggesting that apoptosis has an important role in the resolution process of the newly formed connective tissue [4]. 

The radiographic pattern usually shows multifocal consolidation, mostly peripheral and preferentially affecting the lower zones [9]. Our patient presented extrapulmonary findings like pleural effusions and mediastinal lymph node enlargement, which have been described in a minority of patients [10]. The differential diagnosis is extensive, but OP is essentially a diagnosis of exclusion. Due to the pathological findings, lung biopsy is still the preferred method for establishing the diagnosis [4].

The management of OP is not well established, and treatment options are usually based on general clinical considerations including the time of onset, disease severity, and disease progression [11]. Treatment options described in the literature comprise high doses of corticosteroids, which are usually associated with rapid improvement. However, the optimal initial dose of systemic corticosteroid therapy is not known [8,11].

Some authors suggest an initial prednisone dose of 0.75 to 1 mg/kg per day, based on ideal body weight, for four to eight weeks. If the patient shows improvement, the prednisone dose can be gradually tapered to 0.5 to 0.75 mg/kg per day for four to six weeks [8]. A few case reports have described responses to a macrolide antibiotic, such as clarithromycin 250 mg to 500 mg twice a day, in mild diseases [12,13] Sometimes, it may be necessary to introduce a corticosteroid-sparing agent such as azathioprine when there is a relapse during corticosteroid tapering or side effects with high-dose corticosteroids [8].

Our patient presented complications of treatment with high doses of corticosteroid, but it was not necessary to introduce a corticosteroid-sparing since there was no relapse with the corticosteroid tapering.

## Conclusions

There are multiple etiologies for OP, and it is important to perform a good medical history to find the secondary causes. The majority of cases present with subacute symptoms such as fever, malaise, and a cough that is generally persistent and nonproductive. Patients may also present with dyspnea and weight loss. Differential diagnosis can be challenging, and OP is essentially a diagnosis of exclusion. Radiological findings may be suggestive, but the diagnosis is only confirmed by lung biopsy.

There is no specific treatment modality established. In mild disease, macrolide antibiotics can be used, but in severe disease, corticosteroid therapy is the first line of therapy. In some situations, it may be necessary to introduce a corticosteroid-sparing agent such as azathioprine.
